# Effects of concentrate level and chromium-methionine supplementation on the performance, nutrient digestibility, rumen fermentation, blood metabolites, and meat quality of Tan lambs

**DOI:** 10.5713/ab.20.0802

**Published:** 2021-10-29

**Authors:** Yadong Jin, Yuxiang Zhou

**Affiliations:** 1 Department of Animal Nutrition and Feed Science, College of Agriculture, Ningxia University, Yinchuan, Ningxia Hui Autonomous Region, 750021, China

**Keywords:** Carcass Characteristics, Concentrate Level, Cr-Met, Fatty Acid Composition, Growth Performance, Meat Quality

## Abstract

**Objective:**

This study was conducted to evaluate the effects of concentrate level and chromium-methionine (Cr-Met) supplementation on the growth performance, carcass characteristics, meat quality, and fatty acid composition of Tan lambs.

**Methods:**

Sixty male Tan lambs (21±1.23 kg body weight) fed a finishing diet (concentrate-to-forage ratio: 35:65 [LC group] or 55:45 [HC group]) with daily Cr-Met supplementation (0, 0.75, or 1.50 g) were used in a completely randomized design with a 2×3 factorial arrangement of treatments.

**Results:**

Lambs from the HC group had higher average daily gain, dry matter (DM) digestibility, dressing percentages, leg proportions, intramuscular fat (IMF) contents, and saturated fatty acid levels, but lower feed conversion ratios, globulin (GLB) and total protein (TP) concentrations, shear force, and monounsaturated fatty acid (MUFA) levels (all p<0.05). Cr-Met supplementation increased the DM digestibility, GLB and TP concentrations, rack and loin percentages, and cooking loss, but decreased the IMF contents and leg proportions (all p<0.05). Cr-Met supplementation at 0.75 g/d increased the conjugated linoleic acid (CLA) content in both the HC and LC groups (p<0.01). Significant interactions between the concentrate level and Cr-Met dosage were observed for MUFA (p<0.01) and polyunsaturated fatty acid (PUFA) (p<0.01) levels. Meat from the lambs fed an unsupplemented LC diet presented the highest PUFA and MUFA levels (p<0.01). However, the MUFA and PUFA levels decreased significantly with increasing Cr-Met supplementation levels in the LC group (p<0.01), whereas the opposite trend was seen in the HC group.

**Conclusion:**

The HC diet improved the growth performance of Tan lambs, increased their profitability by increasing leg and rack joint proportions, and improved meat quality by promoting an IMF content that was more visibly acceptable to consumers. Cr-Met supplementation at 0.75 g/d in a HC diet was the best choice and may be economically beneficial.

## INTRODUCTION

Tan sheep are native to China and are mainly distributed in Ningxia Hui Autonomous Region. In recent years, the feeding management for Tan sheep has been changed from grazing to stall-feeding because grazing was banned by the local government. In modern ruminant agriculture, high-concentrate diets are generally used to improve growth performance and maximize economic efficiency. Notably, however, the over feeding of concentrates can also result in increased fat deposition, and negatively affect rumen function, growth performance and meat fatty acid composition [1–5]. Compared with concentrate-based diets, forage-based diets for growing and fattening lambs reduce fat deposition and saturated fatty acid (SFA) content [6], but increase the polyunsaturated fatty acid (PUFA) and conjugated linoleic acid (CLA) levels [5] in lamb meat. Given the increasing demand for red meat production, the optimal concentrate levels for Tan sheep should be determined in order to improve growth performance, and carcass and meat quality and eventually also provide greater economic benefit.

Studies have suggested that metabolic modifiers may be a good choice for improving the growth performance and meat quality of lambs [7,8]. Chromium (Cr) is an essential trace mineral for both humans and animals, and Cr propionate has been approved for use as a food additive for dairy cows [9]. Several studies have suggested that Cr supplementation can improve growth performance [10], while also decreasing fat accumulation [8]. In addition, a small number of studies in pigs have reported that Cr supplementation can improve meat fatty acid composition [11,12]. However, dietary Cr supplementation in sheep has yet to be defined mainly because of the inconsistencies in the reported effects of dietary Cr supplementation on animal production performance. Cr-Met, an amino acid chelate, is cheaper than other types of organic Cr complexes and can be directly absorbed through the intestinal epithelial cell membranes and metabolized without prior digestion [13]. Given these observations, the aim of this study was to investigate the effect of Cr-Met supplementation on growth performance, nutrient digestibility, rumen fermentation, blood parameters, carcass characteristics, and meat quality, in Tan lambs fed with either a low- or high- concentrate diet.

## MATERIALS AND METHODS

### Animal care

All animal procedures were approved by the Laboratory Animal Ethics Committee and the Welfare Committee of Ningxia University (Approval No.: NXU-ACAU-2019-108).

### Experimental design and animal management

This study was conducted at the experimental farm of Ningxia University in Wuzhong City, Ningxia Hui Autonomous Region, from July to October 2019. Sixty male Tan lambs, 5 months old, were grouped by weight in a 2×3 factorial arrangement. After a15-day adaptation period [1,6,14], all the lambs were weighed (average initial body weight [BW]: 21±1.23 kg) and remained in the feeding trial for 65 days. All the lambs had been treated for external and internal parasites with ivermectin (0.2 mg/kg BW, administered subcutaneously; Kaisen Pharmaceutical Co. Ltd, Jiaozuo, China) and albendazole (0.1 g/kg BW, administered orally; Zhonglong Shenli Animal Pharmaceutical Co. Ltd, Hefei, China), respectively, and vaccinated against enterotoxemia, brucellosis, small ruminant lentivirus, and foot and mouth disease before the experiment. Lambs were individually housed in concrete floor pens (1.0×1.5 m) and had *ad libitum* access to water and/or feed throughout the trial. In total, six dietary treatments were devised. Half of the lambs (n = 30) were fed a low-concentrate (LC) diet composed of 35% concentrate and 65% forage and half (n = 30) were fed a high-concentrate (HC) diet composed of 55% concentrate and 45% forage. Cr, as Cr-Met (Availa Cr 1000, Zinpro Inc., Shanghai, China), was then supplemented at three levels within both the HC and LC groups (n = 10 lambs per group) as follows: 0 mg Cr/d, 0.75 mg Cr/d, or 1.50 mg Cr/d. Cr, as Cr-Met, was offered in capsules before the morning feed, and an equivalent number of placebo capsules, without Cr-Met supplementation, were provided to lambs. The ingredients and chemical composition of the diets (based on dry matter [DM] content) are shown in Table 1. The diets were formulated to meet the nutritional requirements recommended by the Chinese Agriculture Industry Standard NY/T 816-2004 [15]. The fatty acid composition of the diets is shown in Table 2. Forage (2 to 3 cm) and concentrate were offered to lambs in two equal portions at 07:00 h and 17:00 h as a totally mixed ration. To determine DM intake (DMI), the weight of feed offered and that of refusals was recorded daily for each lamb before the next morning feeding. In addition, individual samples of feed offered and refusals were collected every week and frozen at −20°C for subsequent determination of DM, crude protein (CP), ether extract, ash, calcium (Ca), phosphorus (P) [16], acid detergent fiber (ADF) and neutral detergent fiber (NDF) contents [17]. The Cr content in the basal diet was analyzed according to Yan et al [14]. Animals were weighed twice at the beginning and end of the experiment before the morning feed to calculate individual average daily gain (ADG) and feed conversion ratio (FCR).

### Nutrient digestibility

Thirty health lambs (n = 5 per treatment) weighing approximately 30 kg were housed individually in metabolism pens (1.3×1.0×1.5 m) to determine the nutrient digestibility. The digestibility trial lasted for 10 days, including 5 days of adaptation. Total feces were collected for each lamb before morning feeding during the last 5 days of the trial. A total of 100 g of each sample was frozen at −20°C for analysis of DM, ADF, and NDF contents. Additionally, another 100 g of each sample was mixed with 20 mL of 10% H_2_SO_4_ and then frozen at −20°C for the determination of CP content. The daily feed and remaining residues were weighed before the morning feeding during the last 5 days of the digestibility trial. At the end of the collection period, the feed and fecal samples obtained over the 5-day trial for each lamb were pooled, and 20% was dried at 65°C for 72 h. The DM and CP content of feeds and feces were determined based on AOAC [16]. The ADF and NDF contents were analyzed according to Van Soest et al [17]. Apparent nutrient digestibility was calculated as a percentage of nutrient intake not recovered in the feces.

### Blood metabolite and hormone levels

Before the morning feed on day 65, blood samples (10 mL) were obtained by jugular puncture and collected into heparin-containing tubes. Blood samples were placed in an incubator with ice bags and then centrifuged at 3,500×g for 15 min at 4°C to separate the plasma. The plasma samples were frozen at −20°C until analysis of metabolite and hormone levels. The plasma concentrations of glucose (GLU), cholesterol (TC), triglycerides, blood urea nitrogen (BUN), total protein (TP), low-density lipoprotein cholesterol, and high-density lipoprotein cholesterol (HDL-C) were analyzed in an automatic biochemical analyzer (Mindray BS-180, Biomedical Electronics Co. Ltd, Shenzhen, China) using the corresponding commercial kits (Mindray, Biomedical Electronics Co. Ltd., China). Plasma insulin (INS) and nonesterified fatty acid concentrations were measured using commercial kits produced by Jingmei (Jingmei Biochemical Reagent Co., Shenzhen, China) according to the manufacturer’s instructions.

### Ruminal fermentation characteristics

At the end of the experiment, approximately 3 h after morning feeding, rumen fluid was collected from five randomly selected lambs from each treatment group using an oral stomach tube (Wuhan Anscitech Farming Technology Co., Ltd, Wuhan, China). Rumen pH was measured using a pH meter (DDSJ-318; Shanghai INESA Scientific Instrument Co., Ltd, Shanghai, China). The rumen fluid samples were subsequently filtered through four layers of sterile cheesecloth. For NH_3_-N analysis, 5 mL of filtered rumen fluid was placed in a centrifuge tube containing 1 mL of H_2_SO_4_ (20 g/L). To determine the volatile fatty acid (VFA) concentration, another 5 mL of filtered rumen fluid was placed in a centrifuge tube containing 1 mL of metaphosphoric acid (250 g/L). The samples for NH_3_-N and VFA analysis were stored at −20°C until analysis.

The rumen fluid VFA concentration was measured using gas chromatography (Agilent, GC7890, Santa Clara, CA, USA) according to Jeon et al [18]. The rumen NH_3_-N concentration was determined using a colorimetric spectrophotometer (UV-759; Shanghai Automation Instrument Co., Ltd, Shanghai, China) based on AOAC [16].

### Slaughter and carcass evaluation

The day before slaughter, all the lambs were weighed before morning feeding at the experimental farm, and 30 lambs were subsequently selected based on the mean BW of each group and marked. The selected lambs were then fasted for 16 h, but with free access to water, until approximately 2 h before slaughter. The lambs were transported to a nearby commercial abattoir where they were reweighed both before slaughtering and after complete bleeding for determination of blood weight. The lambs were slaughtered according to Muslim rites and under veterinary supervision. After complete bleeding, the bodies were skinned and non-carcass components (skin, head, feet, liver, spleen, pluck [total weight of lungs, trachea, and heart], full gastrointestinal tract [GIT], and empty GIT) were weighed. The hot carcass was weighed after all non-carcass components had been removed. Dressing percentages were determined according to the method of Majdoub-Mathlouthi et al [5]. Omental and mesenteric fats were separated from the rumen and intestine, respectively, and weighed. GR measurement, backfat thickness, and loin eye area were measured according to the methods described by Sen et al [19]. The cold carcass was weighed after chilling at 4°C for 24 h. Testes, kidneys, and kidney fat were separated and weighted. Kidney fat, omental fat, and mesenteric fat percentages were expressed relative to cold carcass weight while those of other non-carcass components were expressed relative to pre slaughter BW. Carcasses were then split along the midline into two symmetrical parts. The left half was separated into neck, shoulder, rack, loin, leg, breast and flap according to the specifications of Chinese Agriculture Industry Standard NY/T 1564-200 (technical specification for mutton segmentation) and weighed to calculate their proportions based on the cold carcass weight. The *longissimus thoracis et lumborum* (LTL) muscle was excised from both sides of the carcass. Two LTL samples (50 g for each duplicate) were vacuum-packed and frozen at −20°C until fatty acid analysis, and the remainder was used for meat quality evaluation.

### Meat quality

Drip loss was measured in raw meat after slaughter according to the method described by Sen et al [19]. The pH value of the LTL was determined 24 h (pH_24 h_) after slaughter using Testo-205 pH meter according to the method of Gao et al [20]. Meat color was measured 24 h after slaughter (CIE L*_24 h_ a*_24 h_ b*_24 h_) using a chronometer (MATTHAUS, OPTO-STAR, Eckelsheim, Rheinland-Pfalz, Germany). The intramuscular fat (IMF) content of a 10-g LTL sample was extracted according to the method described by Folch et al [21] and expressed as a percentage of fresh muscle weight. The cooking loss and Warner–Bratzler shear force (WBSF) of the LTL muscle were measured according to Fruet et al [22].

### Fatty acid analysis

The fatty acid composition of the LTL sample was determined based on the method described by Cherif et al [6], using a gas chromatograph (Agilent, GC7890, USA) equipped with a flame ionization detector and a 100% polymethylsiloxane capillary column (100 m×0.25 mm in diameter, 0.25-μm film thickness [HB-88]). The concentration of each fatty acid was expressed as milligrams of fresh muscle weight per gram quantified.

### Statistical analysis

The experiment was performed using a completely randomized design with a 2×3 factorial arrangement of treatments (concentrate level×Cr-Met supplementation dosage). Data were analyzed using the MIXED procedure in SAS (version 8.2; SAS Inst. Inc., Cary, NC, USA). The BW at the beginning of the preparation period was used as a covariate. The linear model was as follows:


$${\text{Y}}_{\text{ijkl}}=\mu +{\text{A}}_{\text{i}}+{\text{B}}_{\text{j}}+{\text{A}}_{\text{i}}\times {\text{B}}_{\text{j}}+{\text{BW}}_{\text{k}}+{\text{e}}_{\text{ijkl}}$$

Where Y_ijkl_ is the observation of the dependent variable; μ is the fixed effect of population mean for the variable; A_i_ is the fixed effect of concentrate-to-forage ratio (i = 2; LC and HC); B_j_ is the fixed effect of the Cr-Met supplementation dosage (j = 3; 0, 0.75, and 1.50 g Cr/d); A_i_×B_j_ is the interaction effect between concentrate-to-forage ratio and Cr-Met supplementation dosage, and e_ijkl_ is the unexplained random effect. The covariance structures (autoregressive [order 1], unstructured, compound symmetry, Toeplitz, and variance component) for the repeated measures model were tested and the structure which best-fitted the model was selected based on the smallest Akaike information criterion value. Duncan’s multiple comparisons test was used to determine the differences among treatment means. Effects were considered significant if p<0.05; 0.05<p<0.10 was considered a tendency.

## RESULTS

### Growth performance and nutrient digestibility

The effects of concentrate level and Cr-Met supplementation on the growth performance and nutrient digestibility of Tan lambs are shown in Table 3. Compared with the LC group, lambs in the HC group had higher final weight, ADG, DMI, and DM digestibility, but lower NDF and ADF digestibility and FCR (all p<0.05). Cr-Met supplementation increased DM digestibility (p = 0.01), with the 1.50 Cr supplementation level eliciting the best results in both the LC diet and HC diet groups.

### Rumen fermentation

The effects of concentrate level and Cr-Met supplementation on rumen fermentation parameters in Tan lambs are shown in Table 4. Compared with those in the LC group, lambs fed the HC diet had lower ruminal pH and acetate-to-propionate ratio, but higher propionate, butyrate and valerate contents (all p<0.01). Additionally, a significant interaction between concentrate level and Cr-Met levels was found for the acetate-to-propionate ratio (p = 0.03). The highest acetate-to-propionate ratio was observed in the LC-0Cr group and the lowest in the HC-0Cr group.

### Blood metabolites

The effects of concentrate level and Cr-Met supplementation on the blood metabolites of Tan lambs are depicted in Table 5. Compared with those in the LC group, lambs in the HC diet group had higher BUN and albumin (ALB) contents, but lower HDL-C, globulin (GLB), and TP contents (all p<0.01). Cr-Met supplementation increased the GLB (p = 0.02) and TP (p = 0.01) levels. For both the LC diet and HC diet groups, the highest GLB and TP levels were seen with the 1.50 Cr supplementation level. Moreover, a significant interaction between the concentrate level and the level of Cr-Met supplementation was found for HDL-C concentration (p<0.01). The LC-0Cr group exhibited the highest HDL-C concentration (p<0.01).

### Carcass characteristics

As shown in Table 6, compared with those in the LC group, lambs fed the HC diet had heavier pre slaughter, hot carcass, and cold carcass weights; greater dressing percentage, eye muscle area, backfat thickness, and IMF content; large leg, blood, skin, and liver proportions; less GIT content; and smaller head, and breast-flap proportions (all p<0.05). Cr-Met supplementation increased the GR measurement and the rack and loin proportions but decreased the IMF content and leg proportion (all p<0.05). Moreover, significant interactions between concentrate level and Cr-Met supplementation levels were observed for blood and breast-flap proportions and IMF content (all p<0.05). The LC-0Cr, HC-1.50Cr, and HC-0Cr groups had the highest breast-flap proportion, blood proportion and IMF content, respectively (p<0.05).

### Meat quality

The effects of concentrate level and Cr-Met supplementation on meat quality are depicted in Table 7. The HC group had lower cooking loss, shear force, pH_24 h_, L*_24 h_, a*_24 h_, and b*_24 h_ relative to the LC diet group (all p<0.05). Cr-Met supplementation increased the cooking loss (p = 0.04) and pH_24 h_ (p<0.01).

### Fatty acid composition

The effects of concentrate level and Cr-Met supplementation on meat fatty acid composition are shown in Table 8. Compared with those in the LC group, lambs fed a HC diet had higher contents of C16:0, C17:0, C18:0, and SFA, but lower contents of C18:1n9c, C18:2n-6, t-9 t-12 C18:2, c-9 c-12 C18:2, c-9 t-11 C18:2, C18:3n-6, C20:3n-6, C20:4n-6, C20:6n-3, MUFA, PUFA and n6, as well as PUFA/SFA and n6/n3 ratios (all p<0.05). Cr-Met supplementation had significant effects on C24:1, C18:2n-6, t-9 t-12 C18:2, c-9 t-11 C18:2, C20:3n-6, C20:5n-3, and n6 levels as well as PUFA/SFA and (rumenic acid) RA/vaccenic acid (VA) ratios (all p<0.05). Furthermore, significant interactions between concentrate level and level of Cr-Met supplementation level were observed for C22:0, C16:1, C18:1n9c, C18:2n-3, t-9 t-12 C18:2, c-10 t-12 C18:2, C18:3n-6, C20:3n-6, C20:5n-3, MUFA, PUFA, n6, n3, and PUFA/SFA ratio (all p<0.05). The LC-0Cr group exhibited the highest C18:1n9c, C18:2n-3, t-9 t-12 C18:2, c-10 t-12 C18:2, C18:3n-6, C20:3n-6, MUFA, PUFA, n6, and n3 contents and PUFA/SFA ratio (all p<0.05).The HC-0.75Cr, HC-0Cr and HC-0.75Cr groups exhibited the highest C22:0, C16:1, and C20:5n-3 levels, respectively (all p<0.05).

## DISCUSSION

### Growth performance

Corn contains a large amount of starch, which can increase the energy available for animal growth. Furthermore, with corn-rich diets, a large amount of starch passes from the rumen unfermented. This starch can be digested and absorbed as GLU after it reaches the small intestine, which reduces the need for hepatic gluconeogenesis and eventually contributes to improve animal growth performance [22]. Thus, an increase in available energy could explain why the ADG was higher and the FCR was lower in the HC group. The higher DMI observed in the HC group in our study was consistent with the results of Majdoub-Mathlouthi et al [5]. The higher DMI may be related to the lower rumen physical fill effect of concentrate compared with that of forage [1], which was consistent with the higher DM digestibility recorded in the HC group.

The effects of Cr supplementation on the growth performance of ruminants reported in previous studies have varied considerably. Domínguez-Vara et al [7] reported a 53 g increase in the ADG, but a 1.1 decrease in the FCR for Rambouillet lambs supplemented with a 0.35 mg Cr/kg proved as Cr-yeast, which was similar to the results obtained in our study. Conversely, Arvizu et al [8] reported that Cr supplementation did not significantly affect growth performance in lambs. Cr is an essential trace mineral for animals. Most studies have reported that Cr supplementation has a positive effect on growth performance under conditions of stress. In the present study, the ADG tended to increase (p = 0.09) with increasing Cr-Met supplementation levels, even though none of the lambs were under stress. According to Lashkari et al [23], Cr supplementation at a supraphysiological dose has a positive effect on the growth performance of growing and fattening ruminants, which could explain why Cr-Met supplementation tended to increase the ADG.

### Nutrient digestibility

DMI was reported to have a positive relationship with fluid passage rate [24]. In our study, ADF and NDF digestibility decreased with increasing concentrate level, which might be related to the increased rate of passage through the GIT of lambs in the HC diet group resulting from the higher DMI. Na et al [25] showed that DM digestibility increased with increasing concentrate-to-forage ratio, which was consistent with our findings. The higher proportion of non-fiber carbohydrates and lower proportion of structural carbohydrates could explain why DM digestibility was higher in lambs fed a HC diet. In our study, Cr-Met supplementation had a significant effect on DM digestibility, which was consistent with the trend for an increase in the ADG with increasing Cr-Met supplementation levels in both the LC and HC groups.

### Ruminal fermentation characteristics

The lower ruminal pH value in the HC group was consistent with that reported by Chen et al [26]. A HC diet contains more nonstructural carbohydrates than a LC diet. These can be rapidly fermented in the rumen, leading to increased lactic acid production and, consequently, a lower ruminal pH. The ruminal pH values of all the groups were within the normal range, indicating that the HC diet did not disrupt rumen homeostasis. Compared with those in the LC group, lambs fed a HC diet had a higher propionate level and a lower acetate-to-propionate ratio, which suggested that the HC diet could provide more available energy for animal production. Reports on the effects of Cr supplementation on rumen fermentation characteristics are scarce. In the present study, we found that Cr-Met supplementation had no significantly effect on pH, NH_3_-N content, or concentrations of individual VFAs. However, supplementation of the HC diet with 0.75 g/d Cr-Met significantly increased the acetic-to-propionate ratio relative to the unsupplemented HC diet. Nevertheless, the daily gain and nutrient digestibility of Tan lambs in the HC-0.75Cr group did not decrease, likely because the small intestine, and not the rumen, is the main site of Cr absorption in ruminants [23].

### Blood metabolites

Jeon et al [18] reported that, in cattle, a high-fiber diet increased the TC concentration, compared with that for a high-starch diet. In our study, the TC concentration showed an increasing trend with decreasing concentrate levels. Sung et al [27] suggested that HDL-C can help prevent artery wall narrowing by transporting excess cholesterol to the liver for excretion. Thus, the similar change trends observed for plasma TC and HDL-C concentrations could explain why lambs in the LC-0Cr group had the highest HDL-C concentration and also why the HDL-C content showed opposing trends between the LC and HC groups with increasing Cr-Met supplementation levels.

The higher BUN and lower TB and GLB contents in the HC group relative to the LC group suggested that the consumption of a HC diet decreased the bioavailability of dietary protein and suppressed the humoral immune response in lambs [18,26]. In addition, Cr-Met supplementation had a positive effect on TB and GLB concentrations, indicating that Cr-Met may improve humoral immunity in lambs. The higher ALB content suggested that the HC diet enhanced both liver function and skeletal development [28].

### Carcass characteristics

In the present study, lambs fed the HC diet had greater pre slaughter weight, as well as hot and cold carcass weight, relative to those fed the LC diet. These results may be related to the higher final weight because all four weight indicators showed a similar trend and were consistent with the results of Majdoub-Mathlouthi et al [5]. Thus, Cr-Met supplementation did not affect the pre slaughter weight or the hot and cold carcass weight of lambs from either the LC or HC group, as the final weight was not affected. The eye muscle area is an important indicator for evaluating muscle development. Animals with greater pre slaughter weight normally exhibit a larger eye muscle area [19], which could explain why concentrate supplementation, but not Cr-Met supplementation, increased the eye muscle area. Several studies have demonstrated that Cr supplementation reduces protein catabolism and enhances protein anabolism by increasing INS activity and reducing ubiquitin mRNA levels, while concomitantly increases the levels of insulin-like growth factor-I and insulin-like growth factor-I receptor [11,29]. This may explain why Cr-Met supplementation increased the value of the GR measurement.

Papi et al [2] reported a 4.9% increase in dressing percentage when the dietary concentrate proportion was increased from 30% to 50%, which was consistent with our findings. This could be explained by the higher non-carcass percentage (48.42% vs 46.35%, respectively), and especially the higher GIT content (19.45% vs 15.09%, respectively), in lambs fed the LC diet compared with that in lambs fed the HC diet. In agreement with our results, Fruet et al [22] found that a lower GIT content led to an increase in dressing percentage. Based on previous studies [5,30], these differences in the proportions of skin, head, liver, and GIT content may be associated with differences in energy supply or forage intake between the HC and LC groups. Our results showed that the HC diet may have had a positive effect on increasing blood proportion in lambs, which was inconsistent with the results reported by Moron-Fuenmayor and Clavero [30], who found that blood proportion was not affected by concentrate supplementation. Moreover, the blood proportion showed a linear increase with increasing Cr-Met supplementation in lambs fed the HC diet, which may be related to the higher Cr contents in the blood [23]. More research is needed to determine the effect of concentrate levels and Cr-Met supplementation on blood proportion owing to the limited results obtained in the present study.

The greater backfat thickness and IMF content reflected an increase in energy uptake by lambs fed the HC diet compared with that in lambs fed the LC diet. These findings were consistent with those reported by Mushi et al [31], who observed an increase in carcass fatness and IMF content in goats when the concentrate level was increased from 33% to 66%. Moreover, Papi et al [2] also found a 3.95-mm increase in backfat thickness in lambs when the concentrate level was increased from 30% to 50%. One study showed that lamb meat with an IMF value ranging from 3.5 to 4.5 was more visibly appealing to consumers [32]. Thus, lambs fed the HC diet or the Cr-Met supplemented HC diet produced more visibly acceptable meat compared with those fed the LC diet. Furthermore, in the present study, a higher concentrate level did not significantly increase kidney fat, omental fat, or mesenteric fat proportions, indicating that the HC diet did not reduce carcass yield. These different responses to concentrate supplementation may be linked to differences in energy substrate uptake into these fat reservoirs.

Studies have demonstrated that Cr supplementation exerts a positive effect on decreasing carcass fatness and increasing carcass leanness [8], which was similar to the results obtained in our study. These benefits of supplemental Cr-Met may be related to its apparent effect on the shifting of energy from adipose to lean tissue [7,23]. According to Arvizu et al [8], fat deposition sites in animals display some differences in insulin sensitivity, while Cr supplementation reduces fat deposition by altering insulin sensitivity at different fat deposition sites. This could explain why Cr-Met supplementation did not affect kidney fat, omental fat, or mesenteric fat, but linearly decreased the IMF content.

Analysis of carcass cut proportions showed that the leg proportion, but not those of the breast and flap, increased with increasing concentrate level. This indicated that increasing the concentrate level may improve the profitability of Tan lambs because of the higher market value of the leg joints [5]. In agreement with our results, Moron-Fuenmayor and Clavero [30] observed a small increase in the leg proportion with increasing concentrate levels. Relatively few studies have investigated the effects of Cr supplementation on carcass cut percentages, especially in lambs. In the present study, we observed a decrease in leg proportion, but a increase in loin and rack proportions, with increasing Cr-Met supplementation levels in both the LC and HC groups. These results may be related to the fact that Cr-Met supplementation reduces leg fat deposition, but increases protein synthesis in the rack and loin muscle [8,33]. In addition, Cr-Met supplementation in the HC diet, especially at the 0.75 g/d level, decreased the neck percentage, which may also contribute to the profitability of Tan lambs because of the lower market price of the neck joints.

### Meat quality

Cooking loss and shear force are strongly affected by fatness, with leaner meat normally having higher values for these parameters compared with fatter meat [34,35]. This is consistent with our results showing that, compared with the HC group, meat from lambs in the LC group had higher cooking loss and shear force values. And, although Cr-Met supplementation did not significantly affect cooking loss or shear force; it nevertheless induced a decrease in IMF content in both the LC and HC groups, which may have occurred because fat variation must attain a certain magnitude to affect cooking loss and shear force [35].

Sullivan and Calkins [36] reported that the meat of Tan lambs fed a HC diet is tender (the WBSF values were smaller than 38.2 N); however, that from lambs fed a LC diet supplemented with Cr-Met was tougher (WBSF values were greater than 45.1 N). Thus, these results (Table 7) suggested that the HC diet (a concentrate-to-forage ratio of 55:45) can improve the tenderness of Tan lambs meat; meanwhile, Cr-Met supplementation in the HC diet did not negatively affect meat tenderness.

Meat color is a major factor by which consumers judge meat quality at the point of purchase. Majdoub-Mathlouthi et al [5] reported that the concentrate level did not affect *longissimus lumborum* meat color. However, Fruet et al [22] observed an increase in lightness (L*) and yellowness (b*) in meat from lambs provided feedlot diets relative to those fed on pasture diets. In contrast, we found that meat color decreased with increasing concentrate level. The lower L*_24 h_ and b*_24 h_ values may be explained by the greater slaughter weight of the lambs in the HC group [5]. Gao et al [20] found that a* decreased with increasing concentrate level, which was consistent with our results.

In the present study, the pH_24 h_ was lower in the HC group than in the LC group. These results are in agreement with those reported by Mushi et al [31] and Gao et al [20]; however, Fruet et al [22] and Majdoub-Mathlouthi et al [5] observed that the pH_24 h_ value was not affected by the concentrate level. According to Mushi et al [31], these variable results may have resulted from between-study differences in the make-up and energy levels of feeds. A high pH_24 h_ is generally associated with a depletion of muscle glycogen. Postmortem, muscle glycogen is degraded into lactate and H^+^, leading to a decrease in the pH of the meat [20]. Muscle glycogen content at slaughter is negatively correlated with muscle pH_24 h_ [20]. Consequently, increased muscle glycogen content resulting from increased concentrate levels or energy supply will eventually reduce muscle pH_24 h_ postmortem. Cr is an integral component of the glucose tolerance factor (GTF) complex, which can potentiate the action of INS [11]. Thus, dietary Cr supplementation can improve GLU utilization, leading to a decrease in muscle glycogen content, which could explain why Cr-Met supplementation led to an increase in muscle pH_24 h_ in the present study. However, the higher pH_24 h_ in Cr-Met-supplemented lambs from the LC group was not within the acceptable range (5.6 to 6.4), which could negatively affect consumer acceptance.

### Fatty acid composition

Total SFA content, especially that of C16:0, C17:0, and C18:0, increased with increasing concentrate level in the present study, which was consistent with the results of Cherif et al [6]. This may be related to the higher C16:0, C17:0, and C18:0 contents in the HC diet relative to the LC diet (Table 2). Compared with the LC diet, the SFA content was higher, whereas those of PUFA and MUFA were lower, in the meat of lambs fed a HC diet. This implied that the HC diet created an optimal rumen pH (6.48 vs 6.73 for the HC and LC groups, respectively) for bacteria that act in the process of biohydrogenation.

According to Wood et al [37], to obtain health benefits, a value equal to 0.4 is recommended for the PUFA/SFA ratio, and lower PUFA/SFA rates can induce an increase in cholesterolemia [5]. Our results (Table 8) showed that the PUFA/SFA ratio in the meat of lambs from the LC group without Cr-Met supplementation was higher (0.42) than that recommended value, whereas Cr-Met supplementation or a HC diet had a positive effect on maintaining the PUFA/SFA ratio at a reasonable level. However, Cr-Met supplementation in the LC diet had a negative effect on the contents of several PUFAs, such as C18:2n-6 and C20:3n-6, which are considered beneficial for human health. In contrast, Cr-Met supplementation in the HC diet tended to increase PUFAs and MUFAs contents. These results indicated that Cr-Met supplementation in HC diets can improve fatty acid composition to a greater extent than Cr-Met supplementation in LC diets.

According to Wood et al [38], an n6/n3 ratio of 4 is recommended for human nutrition. In the present study, the meat of lambs fed a diet with a greater forage content (LC group) had a higher n6/n3 ratio than that of lambs fed a diet with a greater concentrate content (HC group); however, the values for both groups were lower than that recommended for human nutrition, as well as those reported by other studies [4,5,22,37]. These different results indicate that breed and feed systems have a strong effect on fatty acid composition. Meanwhile, Cr-Met supplementation did not affect the n6/n3 ratio.

C18:2 *cis*-9 *trans*-11, also known as RA, comprises more than 87% of total CLA isomers and is critical for human health [39]. In the present study, Cr-Met supplementation had a quadratic effect on RA concentration in both the LC and HC groups. In agreement with our results, Tian et al [11] found that pigs supplemented with Cr-Met displayed a linear increase in RA content, especially at the dosage of 800 μg/kg. Naturally occurring CLA originates is mainly derived from bacterial isomerization and/or the biohydrogenation of dietary PUFAs in the rumen, or through endogenous synthesis *via* stearoyl Co-A desaturase (SCD)-mediated desaturation of *trans*-fatty acids, such as C18:1 *trans*-11, in adipose tissue. Thus, in the present study, the Cr-Met supplementation-related increase in RA content might be a result of desaturation as pigs are monogastric animals. According to Serra et al [39], SCD enzymatic activity in the muscle may be determined by the formula RA/C18:1 *trans*-11 (VA). In our study, the similar quadratic tendency of the RA/VA ratio and RA content could explain why Cr-Met supplementation at the dosage of 0.75 g/d induced the highest RA content in the muscle.

In conclusion, a HC diet (a concentrate-to-forage ratio of 55:45) improved the growth performance, carcass characteristics and meat sensory appeal of Tan lambs, but did not significantly increase kidney fat, omental fat, or mesenteric adipose tissue deposition compared with that for lambs fed a LC diet. The addition of Cr-Met to a HC diet improved fatty acid composition. At 0.75 g/d, Cr-Met supplementation to both the HC and LC diets increased the CLA content. However, Cr-Met supplementation decreased the contents of some MUFAs and PUFAs in lambs fed the LC diet, and had a negative effect on meat quality, including on pH_24 h_ and shear force, and did not promote a significant improvement in the growth performance of Tan lambs.

## Figures and Tables

**Table 1:** Ingredient proportion and chemical composition of the experimental diets (% DM basis)

Items	Experimental diets^1)^	

	LC	HC
Ingredient composition (%)		
Maize silage	33.00	15.00
Alfalfa hay, 15%CP	32.00	30.00
Corn grain	11.24	29.74
Soybean meal, 43%CP	14.00	14.50
Wheat bran	5.00	6.00
Salt	0.76	0.76
Mineral and vitamin premix^2)^	4.00	4.00
Nutrient content		
Dry matter (%)	57.40	71.61
Metabolizable energy^3)^ (MJ/kg)	9.82	10.94
Crude protein (%)	14.50	14.50
Ether extract (%)	3.26	3.30
Ash (%)	6.14	5.28
Calcium (%)	0.44	0.39
Phosphorus (%)	0.26	0.28
NDF (%)	37.91	29.45
ADF(%)	25.06	19.20
Chromium (mg/kg)	0.28	0.24

CP, crude protein; NDF, neutral detergent fiber; ADF, acid detergent fiber.

1) LC and HC refer to diets with forage to concentrate ratios of 65:35, and 45:55, respectively.

2) Premix provided the following for per kg of diet: Vit A 150,000 IU, Vit D_3_ 50,000 IU, Vit E 375 IU, Fe 9,500 mg, Cu 560 mg, Mn 2,000 mg, Se 6.5 mg, I 65 mg, Zn 1 875 mg, Co 25 mg.

3) ME = 0.134DMD+0.235EE+1.23 [40], ME, metabolizable energy; DMD, dry matter digestibility; EE, ether extract.

**Table 2:** Fatty acids composition of the diets

% of total fatty acids	LC^1)^	HC^1)^
C14:0	0.038	0.041
C14:1	0.121	0.112
C15:0	3.098	3.121
C16:0	13.999	14.781
C17:0	0.119	0.121
C16:1	0.226	0.231
C18:0	3.399	3.465
C18:1	25.762	24.989
C18:2n6	47.106	47.217
C18:3n6	3.716	3.444
C18:3n3	2.065	2.132
C20:0	0.351	0.346

1) LC and HC refer to diets with forage to concentrate ratios of 65:35, and 45:55, respectively.

**Table 3:** Effects of concentrate level and Cr-Met supplementation on growth performance and nutrient digestibility of Tan lambs

Items	LC^1)^			HC^1)^			SEM	p-value^2)^		
		
	0 Cr	0.75 Cr	1.50 Cr	0 Cr	0.75 Cr	1.50 Cr		C	Cr	C×Cr
Initial weight (kg)	22.64	21.84	21.78	22.14	22.10	22.64	0.27	0.73	0.86	0.67
Final weight (kg)	30.86	30.40	32.29	34.34	34.70	35.84	0.54	<0.01	0.08	0.64
ADG (g)	126	132	162	188	194	203	6.94	<0.01	0.09	0.56
DMI (g/d)	945	948	953	1,053	1,054	1,041	13.58	<0.01	0.98	0.90
FCR (g DM/g ADG)	7.52	7.47	6.04	5.77	5.48	5.16	0.24	<0.01	0.09	0.46
Apparent digestibility (%)										
DM	58.37	65.70	67.07	66.68	69.70	70.75	1.17	<0.01	0.01	0.43
CP	76.64	76.00	79.27	75.83	76.67	78.16	0.65	0.77	0.28	0.85
NDF	47.21	52.71	56.18	41.27	40.82	42.12	1.70	<0.01	0.39	0.50
ADF	46.73	46.13	51.85	36.73	38.31	42.70	1.45	<0.01	0.13	0.93

SEM, standard error of the means; ADG, average daily gain; DMI, dry matter intake; FCR, feed conversion ratio; DM, dry matter; CP, crude protein; NDF, neutral detergent fiber; ADF, acid detergent fiber.

1) LC and HC refer to diets with forage to concentrate ratios of 65:35, and 45:55, respectively.

2) C, concentrate level; Cr, Cr-Met, supplementation level; C×Cr, interaction between the concentration level and Cr-Met supplementation level.

**Table 4:** Effects of concentrate level and Cr-Met supplementation on rumen fermentation characteristics of Tan lambs

Items	LC^1)^			HC^1)^			SEM	p-value^2)^		
		
	0 Cr	0.75 Cr	1.50 Cr	0 Cr	0.75 Cr	1.50 Cr		C	Cr	C×Cr
pH	6.86	6.80	6.82	6.46	6.47	6.47	0.06	<0.01	0.99	0.98
NH_3_-N (mg/dL)	12.15	14.35	15.18	13.69	14.84	12.02	0.54	0.82	0.75	0.54
VFA (mmol/L)										
Acetate	77.36	75.68	65.75	75.50	77.43	64.42	2.31	0.95	0.11	0.71
Propionate	20.15	21.84	18.01	29.95	23.97	27.00	1.10	<0.01	0.23	0.33
Butyrate	12.02	11.86	10.2	14.21	16.64	13.09	0.64	0.01	0.21	0.60
Isobutyrate	0.90	0.86	0.75	0.93	0.84	0.82	0.03	0.73	0.22	0.89
Valerate	1.02	1.08	0.83	1.47	1.28	1.27	0.06	<0.01	0.20	0.62
Isovalerate	0.92	1.06	0.81	1.10	0.96	0.99	0.05	0.45	0.56	0.60
Acetate:propionate	3.85^a^	3.47^a^	3.61^a^	2.53^c^	3.26^ab^	2.69^bc^	0.12	<0.01	0.48	0.03

SEM, standard error of the means; VFA, volatile fatty acid.

1) LC and HC refer to diets with forage to concentrate ratios of 65:35, and 45:55, respectively.

2) C, concentrate level; Cr, Cr-Met, supplementation level; C×Cr, interaction between the concentration level and Cr-Met supplementation level.

a–c Means that do not have common superscripts significantly differ within the treatments (p<0.05).

**Table 5:** Effects of concentrate level and Cr-Met supplementation on blood metabolites of Tan lambs

Items	LC^1)^			HC^1)^			SEM	p-value^2)^		
		
	0Cr	0.75 Cr	1.50 Cr	0 Cr	0.75 Cr	1.50 Cr		C	Cr	C×Cr
TC (mmol/L)	1.28	1.15	1.20	1.09	1.09	1.28	0.04	0.47	0.45	0.39
TG (mmol/L)	0.35	0.31	0.41	0.37	0.38	0.43	0.02	0.20	0.12	0.68
GLU (mg/dL)	4.14	4.04	4.12	3.74	4.36	4.11	0.08	0.85	0.38	0.28
NEFA (mmol/L)	3.68	3.92	3.79	3.88	4.35	4.50	0.17	0.20	0.57	0.86
INS (μIU/mL)	9.27	7.71	8.04	7.39	8.92	9.30	0.26	0.76	0.50	0.16
LDL-C (mmol/L)	0.46	0.43	0.46	0.40	0.39	0.50	0.03	0.85	0.38	0.28
HDL-C ( mmol/L)	1.00^a^	0.83^b^	0.86^b^	0.66^c^	0.77^bc^	0.85^b^	0.02	<0.01	0.42	<0.01
BUN ( mmol/L)	5.33	5.06	5.70	8.19	7.01	6.86	0.29	<0.01	0.49	0.42
ALB (g/dL)	18.57	19.80	20.64	23.90	22.79	22.34	0.45	<0.01	0.97	0.13
GLB (g/dL)	44.70	45.19	47.54	35.53	36.96	42.39	1.05	<0.01	0.02	0.35
TP (g/L)	63.27	64.99	68.18	58.90	59.75	64.73	0.90	<0.01	0.01	0.85

SEM, standard error of the means; TC, total cholesterol; TG, triglycerides; Glu, glucose; NEFA, nonesterified fatty acid; INS, insulin; LDL-C, low-density lipoprotein cholesterol; HDL-C, high-density lipoprotein cholesterol; BUN, blood urea nitrogen; TP, total protein; ALB, albumin; GLB, globulin; TP, total protein.

1) LC and HC refer to diets with forage to concentrate ratios of 65:35, and 45:55, respectively.

2) C, concentrate level; Cr, Cr-Met, supplementation level; C×Cr, interaction between the concentration level and Cr-Met supplementation level.

a–c Means that do not have common superscripts significantly differ within the treatments (p<0.05).

**Table 6:** Effects of concentrate level and Cr-Met supplementation on carcass characteristics of Tan lambs

Items	LC^1)^			HC^1)^			SEM	p-value^2)^		
		
	0 Cr	0.75 Cr	1.50 Cr	0 Cr	0.75 Cr	1.50 Cr		C	Cr	C×Cr
Pre slaughter weight (kg)	28.74	28.54	30.00	32.16	32.60	33.64	0.50	<0.01	0.29	0.94
Hot carcass weight (kg)	12.32	12.60	13.42	14.78	14.94	15.20	0.28	<0.01	0.33	0.77
Cold carcass weight (kg)	12.16	12.46	13.25	14.44	14.67	14.98	0.27	<0.01	0.28	0.83
Dressing percentage (%)	42.28	43.66	44.07	44.82	45.02	44.57	0.32	0.01	0.39	0.25
Eye muscle area (cm^2^)	12.64	13.19	13.03	15.78	17.46	17.60	0.61	<0.01	0.59	0.89
GR measurement (mm)	12.22	12.55	14.39	12.14	12.45	13.61	0.31	0.09	0.02	0.36
Backfat thickness (mm)	6.86	6.11	5.72	7.49	7.10	7.01	0.17	<0.01	0.11	0.26
Kidney fat (%)	1.42	1.43	1.16	1.56	1.41	1.42	0.08	0.45	0.67	0.73
Intramuscular fat (% of fresh meat)	2.96	2.85	2.83	4.46	4.37	3.65	0.14	<0.01	0.01	0.04
Omental fat (%)	2.36	2.01	2.01	2.44	2.36	2.43	0.11	0.22	0.72	0.84
Mesenteric adipose (%)	2.64	2.55	2.74	2.78	2.72	2.52	0.09	0.60	0.69	0.67
Carcass cuts percentages (%)										
Neck	5.49	6.58	7.84	8.01	4.89	5.65	0.37	0.96	0.15	0.42
Shoulder	19.67	20.40	19.32	18.45	18.55	18.67	0.40	0.08	0.95	0.57
Rack	14.12	15.07	14.93	14.50	15.98	16.96	0.30	0.10	0.02	0.85
Loin	18.23	19.57	19.38	16.37	19.26	19.84	0.33	0.19	<0.01	0.37
Leg	30.63	28.67	27.85	33.55	31.36	29.74	0.44	0.02	<0.01	0.18
Breast and flap	11.87^a^	9.71^bc^	10.67^b^	9.13^c^	9.95^b^	9.13^c^	0.28	<0.01	0.38	<0.01
Non-carcass percentages (%)										
Blood	3.91^a^	3.98^a^	3.60^a^	3.78^a^	4.70^a^	6.01^b^	0.21	<0.01	0.06	<0.01
Skin	8.73	8.04	8.59	9.45	9.63	9.26	0.16	<0.01	0.78	0.39
Head	6.93	6.77	7.13	6.48	6.65	6.74	0.08	0.04	0.36	0.70
Feet	2.18	2.15	2.21	2.19	2.09	2.17	0.02	0.47	0.38	0.86
Pluck	1.91	1.88	1.82	1.90	1.89	1.96	0.02	0.42	0.33	0.45
Liver	1.49	1.58	1.64	1.78	1.69	1.63	0.03	0.02	0.98	0.10
Kidney	0.28	0.27	0.27	0.26	0.29	0.28	0.01	0.96	0.92	0.26
Spleen	0.17	0.16	0.15	0.14	0.19	0.13	0.01	0.71	0.34	0.48
Testis	0.81	0.76	0.82	0.88	0.70	0.85	0.03	0.78	0.24	0.55
Full tail	3.13	3.43	2.45	2.77	3.86	3.81	0.22	0.30	0.43	0.35
GIT content	20.29	19.53	18.53	15.57	15.01	14.69	0.50	<0.01	0.21	0.81
Empty GIT	6.66	6.61	7.09	6.96	6.92	6.31	0.10	0.68	0.73	0.07

SEM, standard error of the means; GIT, gastrointestinal tract.

1) LC and HC refer to diets with forage to concentrate ratios of 65:35, and 45:55, respectively.

2) C, concentrate level; Cr, Cr-Met, supplementation level; C×Cr, interaction between the concentration level and Cr-Met supplementation level.

a–c Means that do not have common superscripts significantly differ within the treatments (p<0.05).

**Table 7:** Effects of concentrate level and Cr-Met supplementation on meat quality characteristics of Tan lambs

Items	LC^1)^			HC^1)^			SEM	p-value^2)^		
		
	0 Cr	0.75 Cr	1.50 Cr	0 Cr	0.75 Cr	1.50 Cr		C	Cr	C×Cr
Cooking loss (%)	49.82	50.56	50.21	47.36	47.49	47.72	0.37	0.01	0.04	0.43
Shear force (N)	44.57	46.10	45.87	32.14	32.63	37.68	1.59	<0.01	0.57	0.11
Drip loss (%)	1.78	1.81	1.50	1.53	1.74	1.32	0.07	0.11	0.36	0.96
pH_24 h_	6.21	6.49	6.50	6.01	6.26	6.28	0.04	<0.01	<0.01	0.99
L*_24 h_	46.13	43.89	45.58	42.34	43.46	41.00	0.53	<0.01	0.57	0.07
a*_24 h_	20.24	21.42	21.33	19.60	19.44	19.16	0.31	<0.01	0.43	0.08
b*_24 h_	9.13	8.58	9.11	8.74	7.76	7.28	0.24	0.02	0.22	0.41

SEM, standard error of the means.

1) LC and HC refer to diets with forage to concentrate ratios of 65:35, and 45:55, respectively.

2) C, concentrate level; Cr, Cr-Met, supplementation level; C×Cr, interaction between the concentration level and Cr-Met supplementation level.

**Table 8:** Effects of concentrate level and Cr-Met supplementation on fatty acid composition (mg/g) of intramuscular fat of longissimus thoracis et lumborum in Tan lambs

Items	LC^1)^			HC^1)^			SEM	p-value^2)^		
		
	0Cr	0.75 Cr	1.50 Cr	0 Cr	0.75 Cr	1.50 Cr		C	Cr	C×Cr
C14:0	1.86	1.95	1.94	1.94	1.92	1.91	0.012	0.78	0.43	0.12
C15:0	0.49	0.51	0.51	0.50	0.50	0.50	0.003	0.26	0.28	0.24
C16:0	14.43	14.73	14.92	15.26	14.99	15.02	0.079	<0.01	0.67	0.06
C17:0	1.89	1.93	1.89	1.97	1.99	1.98	0.013	<0.01	0.48	0.83
C18:0	11.38	12.17	11.71	11.97	12.08	12.35	0.101	0.04	0.14	0.24
C20:0	0.07	0.06	0.07	0.07	0.07	0.07	0.001	0.51	0.08	0.12
C22:0	0.085^ab^	0.084^bc^	0.085^ab^	0.085^ab^	0.086^a^	0.082^c^	0.001	0.66	0.06	0.02
C24:0	0.13	0.12	0.13	0.13	0.13	0.13	0.001	0.28	0.87	0.95
C14:1	0.06	0.07	0.07	0.07	0.07	0.07	0.001	0.92	0.54	0.08
C15:1	1.41	1.45	1.43	1.40	1.40	1.40	0.008	0.07	0.58	0.80
C16:1	1.50^a^	1.53^ab^	1.58^bc^	1.61^c^	1.55^abc^	1.48^a^	0.012	0.59	0.60	<0.01
C20:1	0.09	0.09	0.09	0.09	0.09	0.09	0.001	0.77	0.39	0.89
C24:1	0.222	0.229	0.207	0.215	0.224	0.217	0.003	0.93	0.03	0.16
t-11C18:1	0.58	0.53	0.53	0.53	0.54	0.54	0.006	0.17	0.20	0.06
C18:1n9t	0.03	0.28	0.03	0.02	0.03	0.03	0.001	0.06	0.86	0.11
c-11C18:1	0.02	0.02	0.02	0.02	0.02	0.02	0.001	0.47	0.23	0.09
C18:1n9c	43.33^a^	40.96^b^	39.52^bc^	38.96^c^	39.39^bc^	40.05^bc^	0.347	<0.01	0.09	<0.01
C18:2n-3	7.59^a^	7.24^ab^	7.12^b^	7.09^b^	7.25^ab^	7.44^ab^	0.057	0.60	0.77	0.01
C18:2n-6	0.162	0.152	0.152	0.156	0.146	0.146	0.001	0.02	<0.01	0.93
t-9 t-12 C18:2	2.81^a^	2.60^b^	2.45^c^	2.43^c^	2.45^c^	2.47^bc^	0.030	<0.01	0.01	<0.01
c-9 t-12 C18:2	0.13	0.12	0.12	0.12	0.12	0.13	0.001	0.29	0.66	0.18
c-9 c-12 C18:2	0.073	0.073	0.073	0.068	0.064	0.065	0.001	<0.01	0.67	0.48
c-10 t-12 CLA	0.018^a^	0.016^b^	0.015^b^	0.016^b^	0.016^b^	0.017^ab^	0.001	0.71	0.10	<0.01
c-9 t-11 CLA	0.574	0.605	0.582	0.559	0.600	0.561	0.005	0.04	<0.01	0.29
t-10 c-12 CLA	0.21	0.21	0.21	0.21	0.20	0.20	0.002	0.10	0.84	0.73
C18:3n-6	0.144^a^	0.133^b^	0.127^b^	0.126^b^	0.128^b^	0.131^b^	0.002	0.01	0.14	<0.01
C18:3n-3	0.198	0.178	0.180	0.188	0.188	0.194	0.002	0.24	0.15	0.05
C20:3n-6	0.022^a^	0.021^b^	0.021^bc^	0.020^d^	0.021^bc^	0.020^c^^d^	0.000	<0.01	<0.01	<0.01
C20:4n-6	0.692	0.689	0.682	0.678	0.681	0.659	0.003	0.02	0.12	0.60
C20:5n-3	0.224^ac^	0.228^a^	0.226^a^	0.226^a^	0.237^b^	0.219^c^	0.001	0.38	<0.01	<0.01
C22:6n-3	0.044	0.043	0.042	0.042	0.042	0.041	0.000	0.04	0.07	0.75
SFA^3)^	30.34	31.57	31.25	31.92	31.77	32.04	0.163	<0.01	0.22	0.12
MUFA^4)^	47.26^a^	44.91^b^	43.48^bc^	42.92^c^	43.31^bc^	43.90^bc^	0.344	<0.01	0.08	<0.01
PUFA^5)^	12.88^a^	12.31^b^	12.01^b^	11.93^b^	12.14^b^	12.29^b^	0.075	0.02	0.17	<0.01
PUFA/SFA	0.425^a^	0.390^b^	0.385^bc^	0.374^c^	0.382^bc^	0.384^bc^	0.004	<0.01	0.01	<0.01
n6^6)^	4.03^a^	3.79^b^	3.63^c^	3.60^c^	3.61^c^	3.62^c^	0.034	<0.01	<0.01	<0.01
n3^7)^	8.05^a^	7.69^ab^	7.57^b^	7.55^b^	7.72^ab^	7.89^ab^	0.057	0.64	0.76	0.01
n6/n3	0.50	0.49	0.48	0.48	0.47	0.46	0.004	<0.01	0.18	0.97
RA/VA	0.98	1.14	1.09	1.05	1.11	1.04	0.015	0.97	<0.01	0.10

SEM, standard error of the means; CLA, conjugated linoleic acid; SFA, saturated fatty acids; MUFA, monounsaturated fatty acids, PUFA, polyunsaturated fatty acids; RA, rumen acid = c-9 t-11 CLA; VA, vaccenic acid = t11 C18:1.

1) LC and HC refer to diets with forage to concentrate ratios of 65:35, and 45:55, respectively.

2) C, concentrate level; Cr, Cr-Met, supplementation level; C×Cr, interaction between the concentration level and Cr-Met supplementation level.

3) SFA (C14:0, C15:0, C16:0, C17:0, C18:0, C20:0, C22:0, C24:0).

4) MUFA (C14:1, C15:1, C16:1, C20:1, C24:1; t-11C18:1, c-11C18:1, C18:1n9t, C18:1n9c).

5) PUFA (C18:2n-3, C18:2n-6, C18:3n-3, C18:3n-6, t-9 t-12 C18:2, c-9 t-12 C18:2, c-9 c-12 C18:2, c-9 t-11 C18:2, c-10 t-12 C18:2, t-10 c-12 C18:2, C20:3n-6, C20:4n-6, C20:5n-3, C22:6n-3).

6) n-6 (t-9 t-12 C18:2, c-9 t-12 C18:2, c-9 c-t12 C18:2, C18:2n-6, C18:3n-6, C20:3n-6, C20:4n-6).

7) n-3 (C18:2n-3, C18:3n-3, C20:3n3, C20:5n-3, C22:6n-3).

a–c Means that do not have common superscripts differ with the treatments (p<0.05).

## References

[1] Jacques J, Berthiaume R, Cinq-Mars D (2011). Growth performance and carcass characteristics of Dorset lambs fed different concentrates: Forage ratios or fresh grass. Small Rumin Res.

[2] Papi N, Mostafa-Tehrani A, Amanlou H, Memarian M (2011). Effects of dietary forage-to-concentrate ratios on performance and carcass characteristics of growing fat-tailed lambs. Anim Feed Sci Technol.

[3] Wang Y, Xu L, Liu J, Zhu W, Mao S (2017). A high grain diet dynamically shifted the composition of mucosa-associated microbiota and induced mucosal injuries in the colon of sheep. Front Microbiol.

[4] Aurousseau B, Bauchart D, Calichon E, Micol D, Priolo A (2004). Effect of grass or concentrate feeding systems and rate of growth on triglyceride and phospholipid and their fatty acids in the M. longissimus thoracis of lambs. Meat Sci.

[5] Majdoub-Mathlouthi L, Saïd B, Say A, Kraiem K (2013). Effect of concentrate level and slaughter body weight on growth performances, carcass traits and meat quality of Barbarine lambs fed oat hay based diet. Meat Sci.

[6] Cherif M, Valenti B, Abidi S (2018). Supplementation of Nigella sativa seeds to Barbarine lambs raised on low- or high-concentrate diets: effects on meat fatty acid composition and oxidative stability. Meat Sci.

[7] Domínguez-Vara IA, González-Muñoz SS, Pinos-Rodríguez JM (2009). Effects of feeding selenium-yeast and chromium-yeast to finishing lambs on growth, carcass characteristics, and blood hormones and metabolites. Anim Feed Sci Technol.

[8] Arvizu RR, Dominguez IA, Rubio MS (2011). Effects of genotype, level of supplementation, and organic chromium on growth performance, carcass, and meat traits grazing lambs. Meat Sci.

[9] Spears JW, Lloyd KE, Krafka K (2017). Chromium concentrations in ruminant feed ingredients. J Dairy Sci.

[10] Mousaie A, Valizadeh R, Naserian AA, Heidarpour M, Mehrjerdi HK (2014). Impacts of feeding selenium-methionine and chromium-methionine on performance, serum components, antioxidant status, and physiological responses to transportation stress of Baluchi ewe lambs. Biol Trace Elem Res.

[11] Tian YY, Gong LM, Xue JX, Cao J, Zhang LY (2015). Effects of graded levels of chromium methionine on performance, carcass traits, meat quality, fatty acid profiles of fat, tissue chromium concentrations, and antioxidant status in growing-finishing pigs. Biol Trace Elem Res.

[12] Jin CL, Wang Q, Zhang ZM (2018). Dietary supplementation with pioglitazone hydrochloride and chromium methionine improves growth performance, meat quality, and antioxidant ability in finishing pigs. J Agric Food Chem.

[13] Ohh SJ, Lee JY (2005). Dietary chromium-methionine chelate supplementation and animal performance. Asian-Australas J Anim Sci.

[14] Yan X, Zhang W, Cheng J (2008). Effects of chromium yeast on performance, insulin activity, and lipid metabolism in lambs fed different dietary protein levels. Asian-Australas J Anim Sci.

[15] Institute of Animal Science of CAAS (2004). Feeding standard of meat-producing sheep and goats.

[16] Association of Official Analytical Chemists (AOAC) (1990). Official methods of analysis.

[17] Van Soest PJ, Robertson JB, Lewis BA (1991). Methods for dietary fiber, neutral detergent fiber, and non-starch polysaccharides in relation to animal nutrition. J Dairy Sci.

[18] Jeon S, Jeong S, Lee M (2019). Effects of reducing inclusion rate of roughages by changing roughage sources and concentrate types on intake, growth, rumen fermentation characteristics, and blood parameters of Hanwoo growing cattle (Bos Taurus coreanae). Asian-Australas J Anim Sci.

[19] Sen AR, Santra A, Karim SA (2004). Carcass yield, composition and meat quality attributes of sheep and goat under semiarid conditions. Meat Sci.

[20] Gao X, Wang Z, Miao J (2014). Influence of different production strategies on the stability of color, oxygen consumption and metmyoglobin reducing activity of meat from Ningxia Tan sheep. Meat Sci.

[21] Folch J, Lees M, Sloane-Stanley GH (1957). A simple method for the isolation and purification of total lipids from animal tissues. J Biol Chem.

[22] Fruet APB, Stefanello FS, Rosado AG, de Souza ANM, Tonetto CJ, Nörnberg JL (2016). Whole grains in the finishing of culled ewes in pasture or feedlot: Performance, carcass characteristics and meat quality. Meat Sci.

[23] Lashkari S, Habibian M, Jensen SK (2018). A review on the role of chromium supplementation in ruminant nutrition-effects on productive performance, blood metabolites, antioxidant status, and immunocompetence. Biol Trace Elem Res.

[24] Lindberg JE (1988). Retention times of small feed particles and of water in the gut of dairy goats fed at different levels of intake. J Anim Physiol Anim Nutr.

[25] Na Y, Li DH, Lee SR (2017). Effects of dietary forage-to-concentrate ratio on nutrient digestibility and enteric methane production in growing goats (Capra hircus hircus) and Sika deer (Cervus nippon hortulorum). Asian-Australas J Anim Sci.

[26] Chen GJ, Song SD, Wang BX (2015). Effects of forage:concentrate ratio on growth performance, ruminal fermentation and blood metabolites in housing-feeding yaks. Asian-Australas J Anim Sci.

[27] Sung KI, Ghassemi Nejad J, Hong SM (2015). Effects of forage level and chromium-methionine chelate supplementation on performance, carcass characteristics and blood metabolites in Korean native (Hanwoo) steers. J Anim Sci Technol.

[28] Zhao MD, Di LF, Tang ZY, Jiang W, Li CY (2019). Effect of tannins and cellulase on growth performance, nutrients digestibility, blood profiles, intestinal morphology and carcass characteristics in Hu sheep. Asian-Australas J Anim Sci.

[29] Peng Z, Qiao W, Wang Z (2010). Chromium improves protein deposition through regulating the mRNA levels of IGF-1, IGF-1R, and Ub in rat skeletal muscle cells. Biol Trace Elem Res.

[30] Moron-Fuenmayor OE, Clavero T (1999). The effect of feeding system on carcass characteristics, non-carcass components and retail cut percentages of lambs. Small Rumin Res.

[31] Mushi DE, Safari J, Mtenga LA, Kifaro GC, Eik LO (2009). Effects of concentrate levels on fattening performance, carcass and meat quality attributes of Small East African×Norwegian crossbred goats fed low quality grass hay. Livest Sci.

[32] Lambe NR, McLean KA, Gordon J (2017). Prediction of intramuscular fat content using CT scanning of packaged lamb cuts and relationships with meat eating quality. Meat Sci.

[33] Gardner GE, Smith G, Pethick DW (1998). Effect of chromium chelavite supplementation on the metabolism of glycogen and lipid in adult Merino sheep. Aust J Agric Res.

[34] Mamani-Linares LW, Gallo CB (2014). Meat quality, proximate composition and muscle fatty acid profile of young llamas (Lama glama) supplemented with hay or concentrate during the dry season. Meat Sci.

[35] Ueda Y, Watanabe A, Higuchi M, Shingu H, Kushibiki S, Shinoda M (2007). Effects of intramuscular fat deposition on the beef traits of Japanese Black steers (Wagyu). Anim Sci J.

[36] Sullivan GA, Calkins CR (2011). Ranking beef muscles for Warner-Bratzler shear force and trained sensory panel ratings from published literature. J Food Qual.

[37] Wood JD, Richardson RI, Nute GR (2004). Effects of fatty acids on meat quality: a review. Meat Sci.

[38] Wood JD, Enser M, Fisher AV (2008). Fat deposition, fatty acid composition and meat quality: A review. Meat Sci.

[39] Serra A, Mele M, La Comba F, Conte G, Buccioni A, Secchiari P (2009). Conjugated linoleic acid (CLA) content of meat from three muscles of Massese suckling lambs slaughtered at different weights. Meat Sci.

[40] CSIRO (2007). Nutrient requirements of domesticated ruminants.

